# Potential Compensation among Group I PAK Members in Hindlimb Ischemia and Wound Healing

**DOI:** 10.1371/journal.pone.0112239

**Published:** 2014-11-07

**Authors:** Laila Elsherif, Mehmet Ozler, Mohamed A. Zayed, Jessica H. Shen, Jonathan Chernoff, James E. Faber, Leslie V. Parise

**Affiliations:** 1 Department of Biochemistry and Biophysics, The University of North Carolina at Chapel Hill, Chapel Hill, NC, United States of America; 2 Cancer Biology Program, Fox Chase Cancer Center, Philadelphia, PA, United States of America; 3 Department of Cell Biology and Physiology, The University of North Carolina at Chapel Hill, Chapel Hill, NC, United States of America; 4 McAllister Heart Institute, The University of North Carolina at Chapel Hill, Chapel Hill, NC, United States of America; 5 Lineberger Comprehensive Cancer Center, The University of North Carolina at Chapel Hill, Chapel Hill, NC, United States of America; Medical University Innsbruck, Austria

## Abstract

PAKs are serine/threonine kinases that regulate cytoskeletal dynamics and cell migration. PAK1 is activated by binding to the small EF hand protein, CIB1, or to the Rho GTPases Rac1 or Cdc42. The role of PAK1 in angiogenesis was established based only on *in vitro* studies and its role in angiogenesis *in vivo* has never been examined. Here we tested the hypothesis that PAK1 is an essential regulator of ischemic neovascularization (arteriogenesis and angiogenesis) and wound healing using a global PAK1 knockout mouse. Neovascularization was assessed using unilateral hindlimb ischemia. We found that plantar perfusion, limb use and appearance were not significantly different between 6–8 week old PAK1^−/−^ and PAK1^+/+^ mice throughout the 21-day period following hindlimb ischemia; however a slightly delayed healing was observed in 16 week old PAK1^−/−^ mice. In addition, the wound healing rate, as assessed with an ear punch assay, was unchanged in PAK1^−/−^ mice. Surprisingly, however, we observed a notable increase in PAK2 expression and phosphorylation in ischemic gastrocnemius tissue from PAK1^−/−^ but not PAK1^+/+^ mice. Furthermore, we observed higher levels of activated ERK2, but not AKT, in ischemic and non-ischemic muscle of PAK1^−/−^ mice upon hindlimb ischemic injury. A group I PAK inhibitor, IPA3, significantly inhibited endothelial cell sprouting from aortic rings in both PAK1^−/−^ and PAK1^+/+^ mice, implying that PAK2 is a potential contributor to this process. Taken together, our data indicate that while PAK1 has the potential to contribute to neovascularization and wound healing, PAK2 may functionally compensate when PAK1 is deficient.

## Introduction

PAKs are serine/threonine kinases involved in many biological processes including cell proliferation, motility and angiogenesis [1]–[3]. Our laboratory originally identified a specific and direct regulator of PAK1 function, the small EF hand-containing protein named CIB1 [4]. CIB1 binds to and activates PAK1 independently of small GTPases [4]. Depletion of CIB1 in endothelial cells results in decreased PAK1 activation and impaired endothelial cell function [5], [6], and we have previously reported that CIB1^−/−^ mice exhibit impaired ischemia-induced angiogenesis in retinal and hindlimb tissue concomitant with a decrease in PAK1 activation [5]. Therefore, we hypothesized that CIB1's contribution to neovascularization *in vivo* is mediated at least in part by PAK1.

The group I PAK family, which is comprised of PAKs 1–3, are involved in all major steps of cell migration from cell polarization and formation of actin-rich protrusions, to contraction of the cell body and retraction of the trailing edge [7]–[10]. The diversity of activators and targets of PAKs can potentially explain their involvement in all major steps of cell migration. In addition to small GTPases and CIB1 [4], PAKs are activated by filamin A [11], tyrosine kinases [12], PDK1 [13], sphingolipids [14], and Akt [15]. Group I PAK downstream targets essential to cell migration include LIM kinase-1 [16], MLCK [17], and MLC [18]. Previous reports showed that a decrease in PAK1 activation impairs angiogenesis and tubule formation in human microvascular endothelial cells (HMEC-1) and in a chick chorioallantoic membrane assay [2], [19]. Furthermore, one or more group I PAK members regulates endothelial cell migration mediated by Ang-1, and its activation is necessary for restoring vascular permeability as the last step in the angiogenesis process that prepares nascent vessels for perfusion [20], [21]. PAK1 is also a strong stimulator of proliferation and migration, leading to the aggressive behavior of human cancers [22]–[29]. All of the aforementioned studies have established a relationship between PAK1 function and angiogenesis *in vitro,* thereby predicting a role for PAK1 in angiogenesis *in vivo*. However, PAK1's role in angiogenesis *in vivo* has never been tested. Moreover, the role of PAK1 in angiogenesis cannot be predicted based on the apparently normal appearance of PAK1^−/−^ mice, since angiogenesis defects are often only revealed under stress, such as an ischemic insult [5], [30]–[33].

In the present study, we used the PAK1 global knockout mouse in unilateral hindlimb ischemia (HLI)—a commonly used model to assess neovascularization–and cutaneous wound healing model, to assess chronic PAK1 loss on neovascularization and wound healing. Surprisingly, loss of PAK1 in the mouse did not affect recovery of hindlimb perfusion after femoral artery ligation, which is well known to depend on collateral remodeling (arteriogenesis) and ischemic capillary angiogenesis. Furthermore, wound repair, which is dependent on injury-induced angiogenesis, was unchanged in the absence of PAK1 *in vivo*. These findings suggest that either a lack of PAK1 contribution or another PAK member may compensate for loss of PAK1. We therefore measured the expression of multiple PAK family members in ischemic gastrocnemius tissue and found that PAK2 protein was significantly upregulated in ischemic tissue of PAK1^−/−^ mice. Furthermore, increased levels of activated ERK2 but not ERK1 or AKT were observed in PAK1^−/−^ compared to PAK1^+/+^ mice following acute ischemic injury. Inhibition of both PAK1 and PAK2 using the pharmacological inhibitor of all group I PAKs, IPA3, results in significant reduction in endothelial cell (EC) sprouting from aortic rings. Our results, therefore, suggest a role for upregulation of PAK2 as a compensatory mechanism in ischemic neovascularization and wound healing in a chronic PAK1 knockout mouse model. This compensatory mechanism appears to be acting through the MAPK pathway. Our results, however, do not determine whether PAK1 contributes to neovascularization in wild type mice, since such a role might only be revealed with rapid, acute, inhibition as with a highly selective PAK1 inhibitor that can be given *in vivo*, which is currently unavailable.

## Material and Methods

### Ethics Statement

All experiments were performed in accordance with national guidelines and regulations and were approved by the University of North Carolina Institutional Animal Care and Use Committee (ICUC ID 11–038.0). All surgery was performed under isoflurane anesthesia. Animals were given analgesic post-operatively and all efforts were made to minimize suffering.

### Animals

Generation of PAK1^−/−^ mice was previously described [34]. All mice used in experiments were backcrossed by our laboratory for at least 10 generations to the C57BL/6 background. PAK1^+/+^ littermates were used as PAK^−/−^ controls for all experiments. Mice were genotyped using PCR with a previously published forward and reverse primer set [34]. Animals used were males and females of 2 age groups, 6–8 weeks and 16 weeks with similar body weights.

### Unilateral HLI

Mice underwent a HLI procedure as previously described [35], [36]. Isoflurane (0.8–1.5%) anesthesia was used during the procedure and during plantar scanning with laser Doppler. Animal temperature was maintained at 37±0.5°C during surgery and scanning. To assess neovascularization following a severe form of HLI, ligation of the femoral artery was performed just distal to the inguinal ligament and proximal to the bifurcation of the popliteal artery. The artery was transected between the ligation points; in addition the superficial epigastric artery was ligated (Figure S1 A). The artery was also transected proximal to the caudal epigastric. In addition to the severe form of HLI ischemia, a milder surgery was performed, consisting of ligation and transection between the lateral caudal femoral artery and proximal to the bifurcation of the popliteal artery, plus ligation of the superficial epigastric (Figure S1 B). Hindlimb ischemia experiments were carried out for a total of 21 days, whereas for the examination of MAPK and AKT signaling, the severe form of HLI (Figure S1 A) was performed for 1 hour followed by euthanasia of the animals and tissue harvest. The antibiotic (cefazolin, 50 mg/kg im) and analgesic (buprenorphine, 9.9 mg/kg) were administered following surgery.

### Laser-Doppler perfusion imaging

Measurements of plantar perfusion were obtained before and immediately after surgery (day 0), and on days 3, 7, 14, and 21 after surgery using a scanning laser-Doppler perfusion imager (model LDI2-IR, Moor Instruments, Wilmington, DE) [35]. Imaging was restricted to a region of interest (ROI) in the hind paw. All ROIs were drawn by an investigator blinded to the mouse treatments, and mean velocity within the ROI was normalized to the area of the ROI. Images were analyzed using MoorLDI PC software. Results are expressed as the ratio of ischemic to non-ischemic (I/NI) plantar regions [35].

### Hindlimb use and appearance score

On days 3, 7, 14, and 21 after HLI, a designated score was given for each animal based on hindlimb use using the following criteria: 0 =  normal; 1 =  no toe flexion; 2 =  no plantar flexion; and 3 =  foot dragging [37], [38]. In addition, foot appearance was also scored using the following criteria: 0 =  normal; 1–5 =  cyanosis or loss of nail(s), dependent on the number of nails affected; 6–10 =  partial or complete atrophy of digit(s), dependent on the number of digits affected; 11 =  and partial atrophy of the forefoot [37]. Higher use and appearance scores correspond to worse outcomes.

### Aortic EC sprouting on Matrigel

Aortic rings from PAK1^−/−^ and PAK1^+/+^ mice were dissected, embedded in growth-factor reduced Matrigel matrix and cultured in 2.5% FBS as previously described [5], [39]. EC sprout number was quantified using phase contrast at days 3 and 5 following embedding. Numbering and counting of sprouts was performed according to a method described by Baker et al. [39]. IPA3 (30 µM) was added to culture media starting at day 0 and for a total of 5 days.

### Ear wound assay

Using a metal ear punch (Harvard Apparatus) a 2.0 mm hole was made in the ears of PAK1^−/−^ and wild type PAK1^+/+^ mice [40]. For imaging of the ear, mice were anesthetized with isoflurane and placed on a flat surface with the ear placed between 2 glass slides. Images were obtained on a Leica-Wild M420 microscope. ImageJ was used to calculate ear-wound diameter on days 1, 3, 7, 14, 21 and 28 after the ear punch. Results are expressed as the ratio of (Area _day x_/Area _day 0_) × 100. On day 28, mice were anesthetized and the ears were fixed with formalin, sectioned and stained with Masson's trichrome.

### Western blot and antibodies

Gastrocnemius muscle from the left (non-ischemic) and right (ischemic) hindlimbs was excised on day 21 after surgery, flash frozen in liquid nitrogen and stored at −80°C until ready for tissue homogenization and processing. The following antibodies were purchased from Cell Signaling Technology, Danvers, MA: PAK1 (#2602), PAK2 (#2608), pPAK2 (Ser20) (#2607), PAK3 (#2609), pan AKT (#4691) phospho-ERK1/2 (Thr202/Tyr204) (#4377), phosphor-AKT (S473) (#9271) and (T308) (#4056). GAPDH antibody (sc-25778) was purchased from Santa Cruz Biotechnologies, Santa Cruz, CA. HRP-conjugated secondary antibodies were purchased from GE Healthcare, Waukesha, WI.

### Statistical Analysis

Data were subjected to 2-way ANOVA or the Student *t* test.

## Results

### The effect of PAK1 loss on neovascularization following ischemic injury

**Figure 1:**
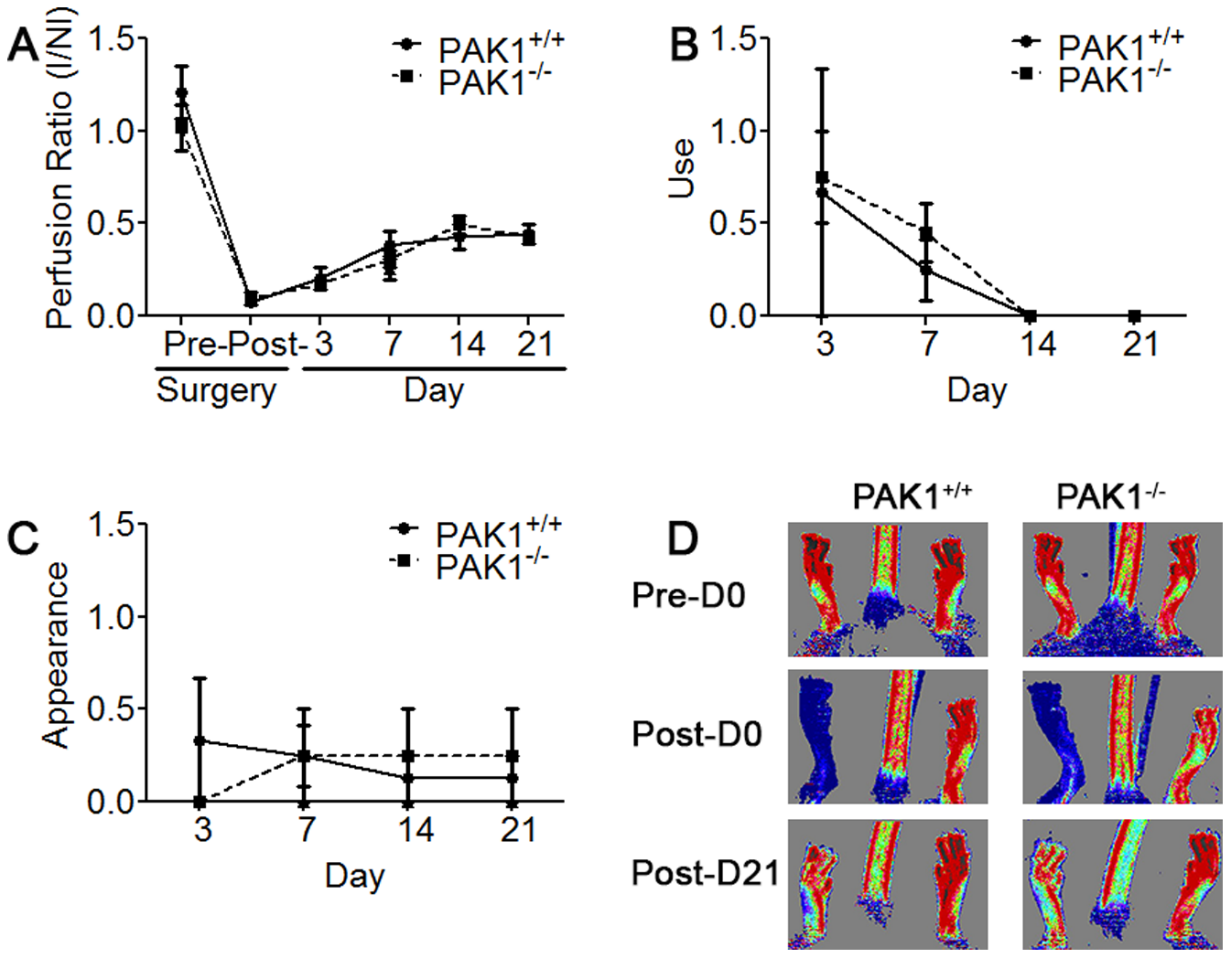
PAK1^−/−^ plantar perfusion and function in model of severe HLI in 6–8 week-old mice is similar to PAK1^+/+^ mice. A) I/NI (Ischemic/Non-Ischemic) plantar perfusion ratio is comparable between PAK1^−/−^ and PAK1^+/+^ mice as measured by laser Doppler imaging from day 0, immediately after HLI surgery, and throughout the 21 days after HLI surgery. B) Limb use score was determined as described in Materials and Methods and is equivalent between groups, where a higher score is observed within the first 2 weeks after surgery and decreased significantly thereafter, and indicating recovery of limb function. C) Appearance scores of PAK1^+/+^ and PAK1^−/−^ mice were not statistically significant. D) Laser Doppler images obtained immediately following HLI surgery and on day 21 reflect a lack of difference in perfusion between PAK1^+/+^ (N = 8) and PAK^−/−^ (N = 11) mice.

**Figure 2:**
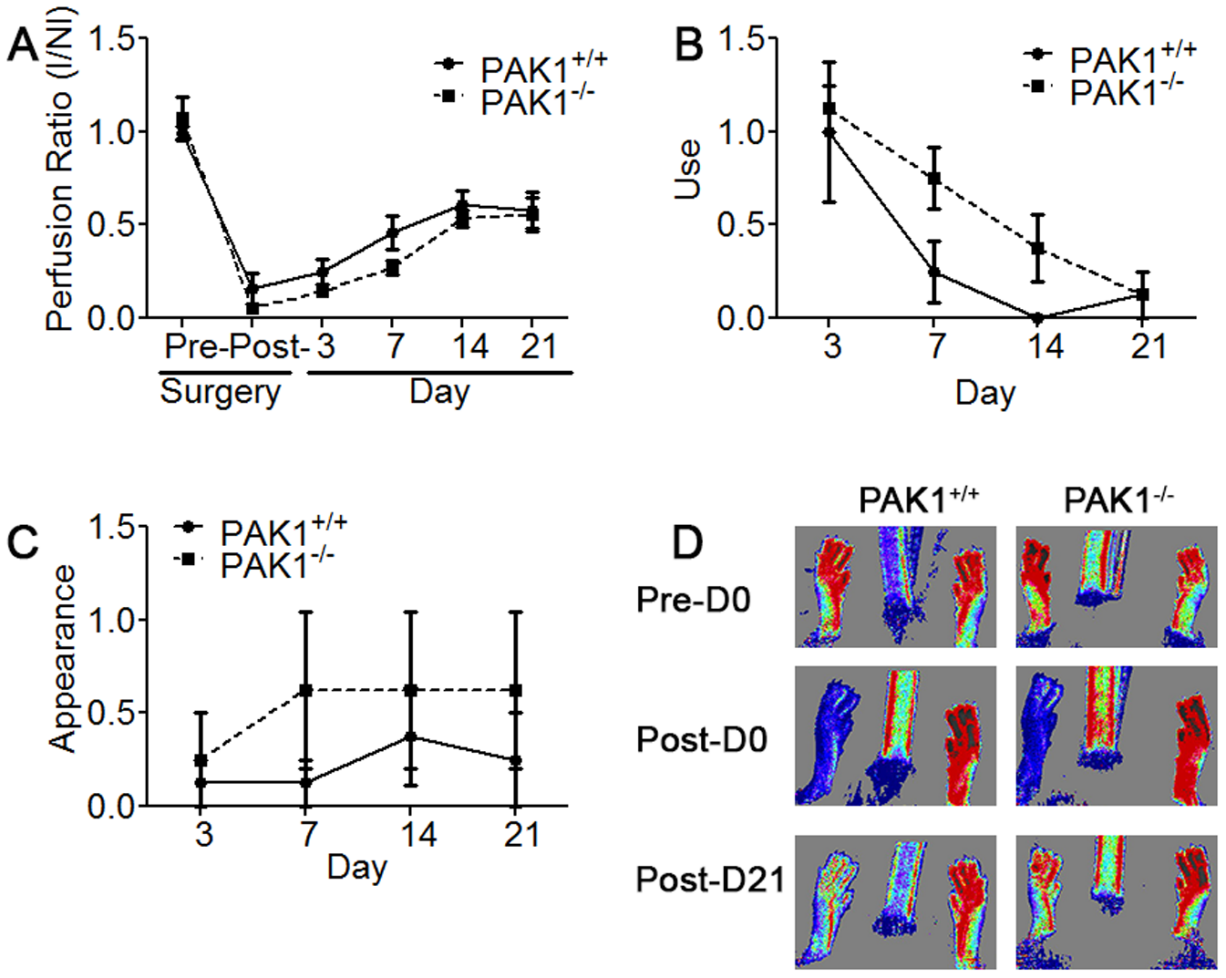
PAK1^−/−^ plantar perfusion and limb function following severe HLI in 16 week-old mice is slightly impaired compared to PAK1^+/+^ mice, suggesting mild impairment in neovascularization in the absence of PAK1. A) I/NI foot perfusion ratio is similar between PAK1+/+ and PAK1−/− mice immediately following surgery, but starts to diverge by days 3 and 7 days (B–C). Worse use and appearance scores in PAK1^−/−^ mice on days 7 and 14 reflect the overall impaired limb function due to impaired neovascularization. D) Laser Doppler images obtained on day 0 pre- and post-surgery, and on day 21 showing similar perfusion between PAK1^−/−^ (N = 8) and PAK1^+/+^ (N = 8) mice.

To assess the contribution of PAK1 to neovascularization, we used both mild and severe forms of HLI that induce ischemia to the muscle tissue supplied by the femoral artery. The severe form of HLI causes more ischemic damage than the mild form, especially in certain mouse strains resistant to ischemic injury such as C57BL6, which is the genetic background of the PAK1^−/−^ and PAK1^+/+^ mice. The designation of mild and severe HLI is described in the Material and Methods section, schematically presented in Figure S1, and is related to the location of artery ligation and transection. Initially we examined the effects of mild HLI on 6–8 week old PAK1^−/−^ mice and found that perfusion immediately following ligation was not different between PAK1^−/−^ and PAK1^+/+^ mice. The level of perfusion immediately following the surgery, which is primarily dependent on the number and average diameter of the native collaterals in the thigh, was not different (Figure S2). This indicates that PAK1 is not involved in collaterogenesis, a process that occurs during development. In addition, there was no significant difference in perfusion and use and appearance scores between PAK1^−/−^ and PAK1^+/+^ mice throughout 21 days (Figure S2). Furthermore, 6–8 week-old PAK1^−/−^ mice subjected to severe HLI showed no differences compared to PAK1^+/+^ mice (Figure 1). By contrast examination of 16 week old PAK1^−/−^ mice revealed a slight–albeit not significant–worsening in recovery of perfusion (Figure 2A and 2D) and use and appearance scores (Figure 2B–C) in PAK1^−/−^ compared to PAK1^+/+^ mice. This trend appeared between days three and seven post-surgery and was no longer evident by day 14 and thereafter.

### Protein expression of other group I PAK kinases in ischemic tissue

**Figure 3:**
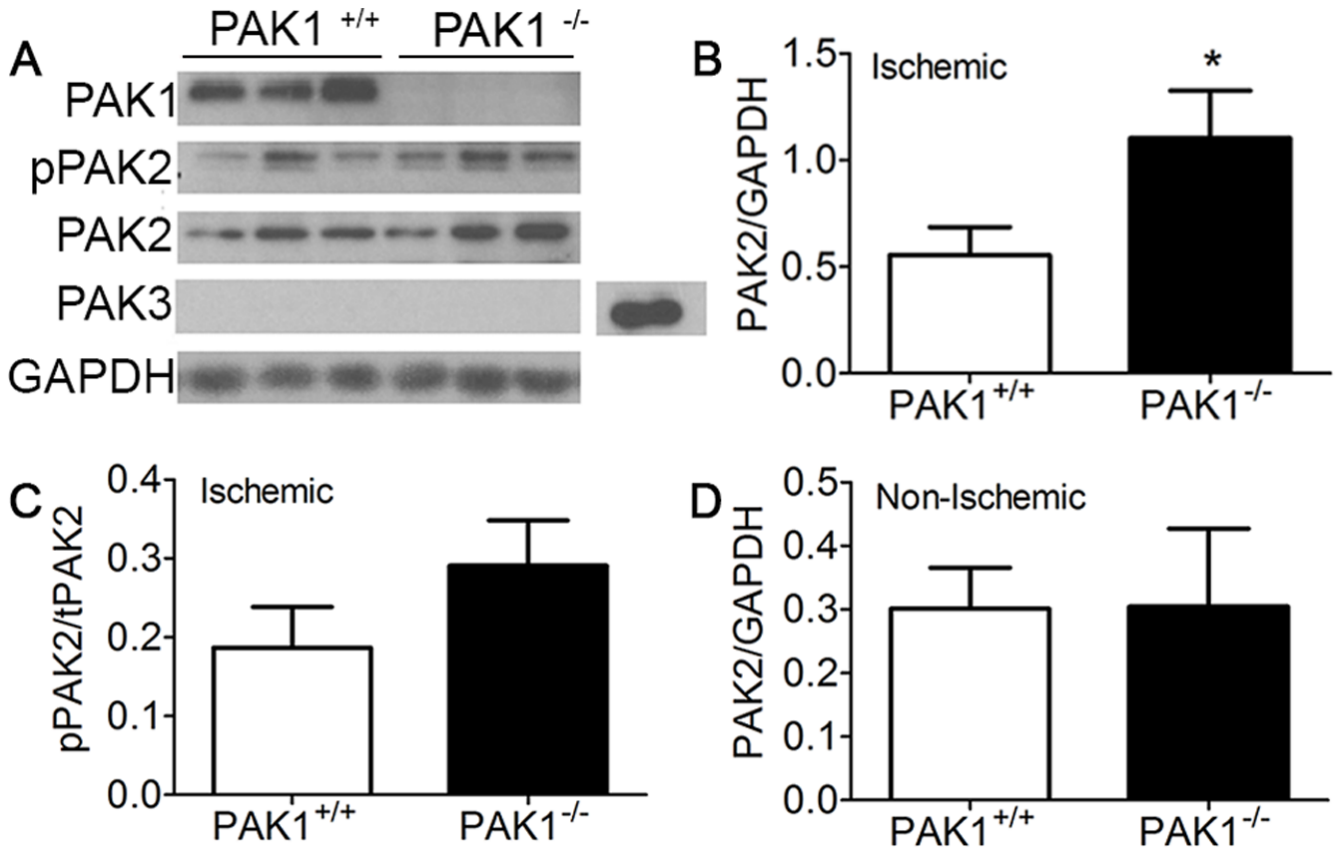
Increased protein expression and phosphorylation of PAK2 in ischemic gastrocnemius muscle in PAK1^−/−^ compared to PAK1^+/+^ mice. A) Western blots showing upregulated PAK2 expression and a trend towards increased phospho-PAK2 in PAK1^−/−^ mice (lanes represent samples from 3 different mice). We did not observe expression of PAK3 in gastrocnemius tissue; however, abundant PAK3 expression is found in mouse brain tissue as can be seen from a positive control sample in lane 7 of the PAK3 blot. B–C) Densitometry analysis reveals a 2-fold increase in PAK2 expression in PAK1^−/−^ compared to PAK1^+/+^ mice, normalized to GAPDH as a loading control, and a concomitant increase in phospho-PAK2 relative to total PAK2. *indicates p≤0.05 using Student's T-test, n = 6. D) Densitometric analysis of total PAK2 protein expression in *non-ischemic* muscle did not reveal a change in PAK1^+/+^ versus PAK1^−/−^ mice.

Group I PAKs have structural and functional similarities, as well as overlapping tissue expression. To explore potential PAK1 compensatory mechanisms we evaluated expression of PAK2 and PAK3 in ischemic gastrocnemius tissue from mice subjected to HLI at day 21 (Figure 3A–B). A two-fold increase in PAK2 protein expression was observed in the ischemic hind-limb gastrocnemius muscle tissue from PAK1^−/−^ compared to PAK1^+/+^ mice (n = 6, Student's T-test, p = 0.03). PAK3 protein expression was not detected in hind-limb tissue, in agreement with reports demonstrating that PAK3 is expressed only in mouse brain tissue (Figure 3A) [41]. Surprisingly the increase in PAK2 expression was observed only in ischemic tissue whereas non-ischemic tissue from PAK1^−/−^ and PAK1^+/+^ mice expressed similar levels of PAK2 (Figure 3B vs 3D). PAK2 activation was determined by the ratio of phosphorylated (p) PAK2 to total PAK2, and although pPAK2 levels also trended towards an increase in PAK1^−/−^ mice compared to PAK1^+/+^ mice, the relative difference was not statistically significant (Figure 3C). These results suggest that ischemia-specific upregulation of PAK2 expression is a potential compensatory mechanism in the chronic absence of PAK1.

### ERK1/2 but not AKT activation is involved in the potential compensatory effect during HLI in PAK1^−/−^ mice

**Figure 4:**
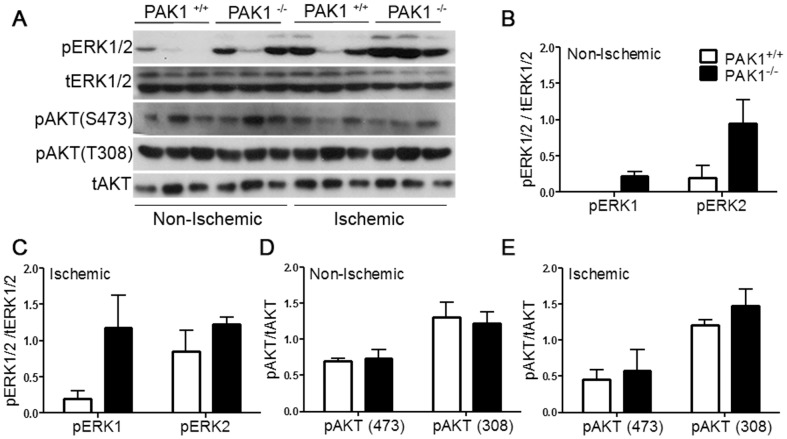
A) A notable activation of ERK1/2 but not AKT is observed in PAK1−/− gastrocnemius tissue one hour after HLI surgery. Western blotting analysis of PAK1^−/−^ non-ischemic and ischemic gastrocnemius muscle shows enhanced phosphorylation of ERK1/2 on Thr202/Tyr204 compared to PAK1^+/+^ tissue, whereas phosphorylation of AKT was unchanged. Lanes are grouped into Non-Ischemic and Ischemic from either PAK1^+/+^ and PAK1^−/−^ mice. B and D) Densitometric analysis of non-ischemic tissue from PAK1^+/+^ and PAK1^−/−^ show an increase in pERK1/2 activation (p = 0.03 and 0.06 for pERK1 and pERK2 respectively, Student's T-test) whereas a difference in pAKT activation is not observed. C and E) In ischemic tissue a trend towards an increase in pERK1 (p = 0.08, Student's T-test) but not pERK2 or pAKT in PAK1^−/−^ compared to control PAK1^+/+^ was observed. A total of 3 PAK1^+/+^ and PAK1^−/−^ mice were analyzed. Blots for pERK1/2 and pAKT were normalized to tERK1/2 and tAKT respectively.

Several studies have shown that PAKs are involved in the ERK-signaling cascade by phosphorylating the ERK upstream molecules, c-Raf and MEK1 [42], [43]. PAKs also serve a scaffolding function for both c-Raf/MEK1 and AKT/PDK1 complexes [44], [45]. Both ERK and AKT signaling pathways are linked to endothelial cell proliferation, migration, and to the angiogenesis process in general [46]–[48]. We therefore opted to examine ERK1/2 and AKT activation in the gastrocnemius muscle of ischemic and non-ischemic PAK1^−/−^ and control mice after one hour of HLI onset. We observed similar total ERK and AKT protein levels in PAK1^−/−^ and PAK1^+/+^ gastrocnemius muscle; however activation of ERK1/2 (Thr202/Tyr204) was observed in non-ischemic tissue from PAK1^−/−^ tissue (Figure 4). In contrast, changes in AKT activation, as assessed by the phosphorylation of S473 and T308, were not observed.

### Effect of inhibition of all group I PAK kinases on EC sprouting from aortic rings

**Figure 5:**
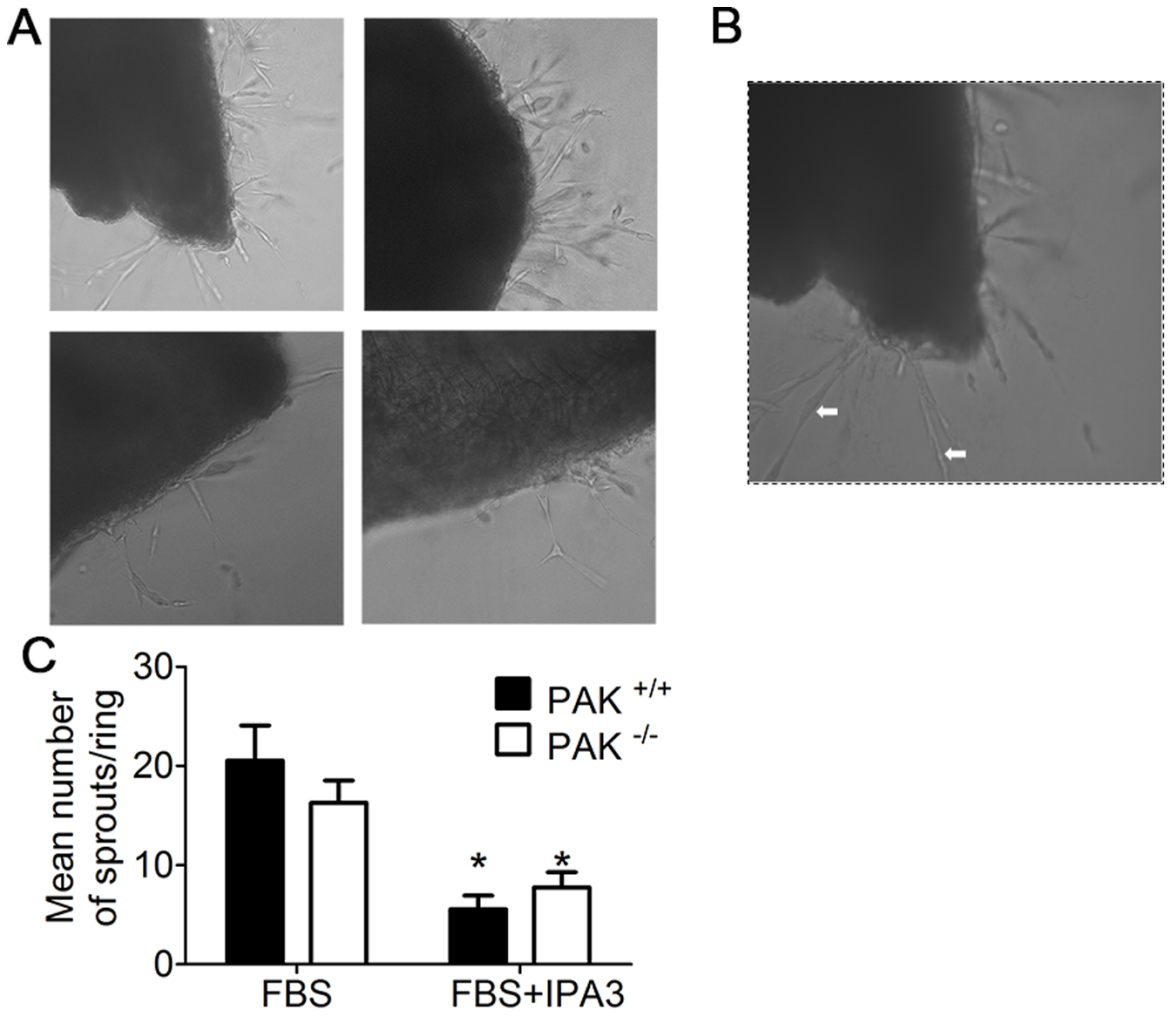
Marked reduction in aortic EC sprouting following IPA3 treatment. A) Similar EC sprouting is observed in PAK1^−/−^ and PAK^+/+^ aortic rings cultured for 5 days in growth media supplemented with FBS, whereas a notable reduction in sprouting was observed following 30 µM IPA3 treatment. B) Magnification of the selected region showing EC sprouts (white arrows). C) Quantification of EC sprouts shows less EC sprouts in PAK1^+/+^ and PAK1^−/−^ with IPA3 treatment. Data points represent the average values ±SEM from 3–9 rings from 3–4 mice per group; * denotes p<0.05 compared to FBS alone.

To determine if other PAK1 family members (e.g. PAK2) compensated for PAK1 loss in another assay of endothelial function, we used aortic ring EC sprouting assays. An equal numbers of EC sprouts were observed in PAK1^+/+^ and PAK1^−/−^ aortic rings (Figure 5A) and treatment with IPA3, an allosteric inhibitor of group I PAKs, significantly inhibited sprout formation in both sets of aortic rings (p = 0.0001, two-way ANOVA) (Figure 5C). These data suggest that both PAK1 and PAK2 are essential for EC sprouting and potentially neovascularization.

### Effect of PAK1 loss on cutaneous wound healing

**Figure 6:**
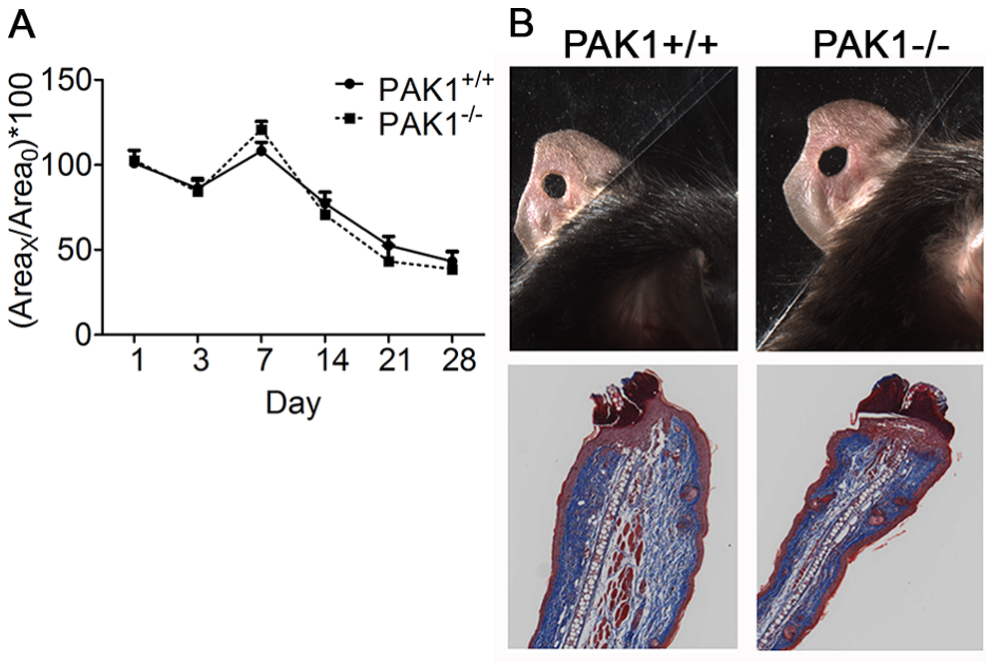
Unimpaired repair of cutaneous ear wounds in PAK1^−/−^ mice. A) Recovery of 2.0 mm ear punch wounds is represented by (Area _day X/_Area _day 0_) ×100. There was a notably larger but not statistically significant wound diameter in PAK1^−/−^ mice compared to PAK1^+/+^ mice on day 7. B) The wound edge was stained with Mason's trichrome and shows a very similar wound closure between the two groups by day 28. n = 3 for both PAK1^+/+^ and PAK1^−/−^ groups.

Ear wound assays are used to assess the migration and function of fibroblasts, macrophages, epithelial cells and subsequent capacity for wound repair and injury-induced capillary sprouting/angiogenesis. In addition to its function in EC motility and function, PAK1 has been shown to affect the function of some of the aforementioned cell types involved in wound healing [49]–[51]; therefore, we sought to determine the *in vivo* effect of PAK1 on cutaneous wound healing. The pattern of ear wound closure following injury in PAK1^−/−^ and PAK1^+/+^ mice was in agreement with published work where wound diameter increases slightly on day 7 and decreases thereafter; however, complete closure is not achieved. Ear wound diameter decreased similarly to 50% by day 28 in both PAK1^−/−^ and PAK1^+/+^ mice (Figure 6A). Very similar tissue architecture was observed in the two groups by day 28 as shown using Mason's trichrome stain (Figure 6B).

## Discussion

We originally hypothesized that loss of PAK1 would impair ischemia-induced neovascularization based on our studies of the CIB1/PAK1 signaling pathway [4], [5]. In addition, PAK1 is intimately linked to proper function and migration of a variety of cell types such as macrophages [50], fibroblasts [49], endothelial cells [1], [2] and tumor cells [22]. We therefore sought to examine the effect of chronic PAK1 loss on: 1) neovascularization, which is dependent on endothelial cell migration and growth; and 2) cutaneous wound healing, which involves migration of epithelial cells and fibroblasts. Our results suggest that the ubiquitous PAK2 is likely to play an important compensatory role in neovascularization in the absence of PAK1 through activation of the ERK-cascade. These results do not necessarily speak to a role for PAK1 in ischemia-induced neovascularization *per se*, since such a role might be revealed upon more acute PAK1 inhibition or depletion. A PAK1-specific pharmacologic inhibitor is not currently available. Our results do indicate, however, that chronic PAK1 absence as in traditional knockout mice, results in elevated PAK2 expression during ischemic injury that could provide functional compensation to preserve tissue perfusion and function. Inhibition of EC sprouting with the group I PAK inhibitor IPA3 further supports a role for PAK2 in sprouting angiogenesis.

Functional compensation between group I PAK members is not surprising given that group 1 PAKs share 80–90% sequence homology in their kinase domain and 88% homology in their p-21 binding domains (PBD) [41]. The signaling function of PAKs can be independent of their kinase activity; thus, a group I member can potentially compensate for the loss of PAK1 by activating downstream targets or by acting as a scaffolding protein in a signaling complex [52], [53]. However, the loss of PAK2 in a traditional knockout mouse results in embryonic lethality due at least in part to improper vessel formation, suggesting the indispensability of PAK2 in endothelial cell function [54]. Our data are the first to suggest potential compensation between PAK1 and PAK2 in neovascularization. However, redundant functions between other PAK members have been reported. Functional redundancy between PAK1 and PAK3 is suggested by data from individual knock-out animals [55]. PAK3 mutations in humans leads to nonsyndromic mental retardation characterized by selective cognition deficits [56], [57]. Loss of PAK3 in mice has no effect on brain or spine morphology and the mice have normal basal synaptic strength; however, they have a deficit in the late phase of long-term potentiation (L-LTP) [55]. This phenotype is closely related to that seen in PAK1^−/−^ mice [58]. Loss of both PAK1 and PAK3 results in learning and memory deficits with hyperactive behavior and lack of complexity in neuronal morphology suggesting functional redundancy between PAK1 and PAK3 [59]. Furthermore among group II PAKs, PAK5 and PAK6 appear to have overlapping functions in the development of the nervous system, whereby separate knockout of these individual genes does not result in a significant phenotype but PAK5/6 double knockout mice present with deficits in learning as well as locomotor activity [60], [61].

Our results show a slightly reduced recovery of perfusion and worse hindlimb ischemic appearance and functional use in PAK1^−/−^ mice at 16 weeks of age, compared to 6–8 week-old mice. Impaired angiogenesis and collateral remodeling in ischemia has been reported previously and linked to several cellular and extracellular mechanisms [62]–[64]. Aging endothelial cells have a reduced capacity for NO production and therefore enhanced sensitivity to apoptotic cell death [65], [66]. Furthermore, decreased levels of certain miRNAs such as miRNA-217 and miRNA-146a have been demonstrated to modulate senescence of endothelial cells *in vitro* [67], [68]. Extracellular factors modulating angiogenesis in aging involve increased expression of TIMP-2 and altered composition of the extracellular matrix [69]. A decrease in the potent growth factors VEGF [62], [70] and TGF-β [71] and an increase in thrombospondin-2 [72] in aging endothelium affects endothelial cell proliferation, migration, and matrix formation. In studies using aged animals, reduced neovascularization capacity was reported following stress or injury to the endothelium [70], [73] as well as in the non-injured endothelium [62]. The neovascularization changes that we observe in slightly older PAK1^−/−^ mice are potentially due to several of the abovementioned factors exacerbated by the absence of PAK1, not only in endothelial cells but also in auxiliary cells such as smooth muscle cells, fibroblasts and immune cells where PAK1 might be indispensable and where PAK2 cannot substitute for its function Interestingly, PAK1 and PAK2 appear to participate in distinct signaling cascades and in some cases play opposing roles in mast [74], breast [75], and prostate [76] carcinoma cells. Whereas PAK1 is a positive modulator of mast cell degranulation through regulation of calcium influx [34], PAK2 is a negative regulator of degranulation, and independent of calcium influx [74]. Furthermore PAK1 and PAK2 appear to have opposing effects on modulating the phosphorylation of MLC and focal adhesions in tumor cells [75]. PAK1 appears to have a pro-adhesion, -migration and -proliferation response whereas PAK2 appears to inhibit these processes.

As mentioned above, PAKs are involved in the activation of the canonical ERK-signaling cascade, which has long been recognized as essential for angiogenesis [77]. Several studies including our own link PAK1 to ERK1/2 [5], [44], [50]. Loss of PAK1 results in downregulation of ERK activity with negative effects on cell motility/migration [29], [78], and proliferation [79]. Furthermore, PAK1 activation induced by adhesion to matrix proteins activates the MAPK signaling cascade and is thought to be a convergence site between integrins and growth factor signaling [80], [81]. However few reports exist on the role of PAK2 in MAPK-signaling and even fewer reports examine the functional significance of PAK2/MAPK signaling. Among these studies are ones examining the role of PAK2 in hematopoietic stem cell function through its phosphorylation of ERK [82] Specifically PAK2 is an essential effector of TGFβ signaling and ERK-mediated transcriptional response [83]. Our results suggest in accordance with some of these previous studies that PAK2 can potentially lead to ERK activation to modulate the physiological angiogenesis response.

Limitations of this investigation stem from the presence of compensatory mechanisms that preclude studying the effect of PAK1 *in vivo* on physiological processes that depend on normal cell migration. One way to circumvent this problem is to generate a double inducible, conditional knockout mouse lacking PAK1 in addition to PAK2, where selectivity for time and location of gene knockdown can be controlled by the investigator. A conditional knockout approach would circumvent the difficulty of deletion of a gene essential for mouse development and allow the examination of gene products in the adult mouse under stress conditions. In addition, this strategy would permit investigation of the specific cell type responsible for the phenotype. However, even with this strategy, compensation is still possible if altered gene expression occurs quickly. With the present study we are reminded of the outstanding ability of biological systems to adjust to environmental and genetic challenges. Systems perturbations that target vital components to development or response to injury will perturb the system; however, genetic redundancy can completely mask these changes. Our study also sheds light on the discord often encountered between conclusions drawn based on studies done *in vitro* and those ascertained from testing in complex organisms. The discord is partly due to an incomplete but growing knowledge of key players and mechanisms of ischemia and neovascularization. The advancement in targeted gene deletion technology and/or targeted pharmacological inhibitors has the potential to allow us to elucidate these mechanisms and identify all the important players throughout the different stages of neovascularization.

## Supporting Information

Figure S1 **Schematic of surgical procedures used in the study.** φ denotes ligation sites and ↔ denotes cutting sites. A) Severe HLI model involves ligation of the femoral artery distal to the inguinal ligament and proximal to the bifurcation of the popliteal artery. The artery was transected between the ligation points; in addition the superficial epigastric artery was ligated. B) Mild HLI model consisting of ligation and transection between the lateral caudal femoral artery and proximal to the bifurcation of the popliteal artery, plus ligation of the superficial epigastric.
(TIF)

Figure S2 **PAK1^−/−^ neovascularization is unimpaired following mild HLI.** A and D) I/NI perfusion ratios are almost identical after mild HLI in PAK1^+/+^ and PAK1^−/−^ mice. B–C) Use and appearance scores that are not statistically different are in agreement with the perfusion ratio observation and confirm the lack of difference in ischemic limb function between PAK1^−/−^ and PAK1^+/+^ mice (n = 7 for PAK1^+/+^ and n = 10 for PAK1^−/−^).
(TIF)
