# Supplementary figures and images for: Potential Compensation among Group I PAK Members in Hindlimb Ischemia and Wound Healing
Source: PLoS One. 2014 Nov 7;9(11):e112239. doi: 10.1371/journal.pone.0112239 (PMC4224450; doi:10.1371/journal.pone.0112239)

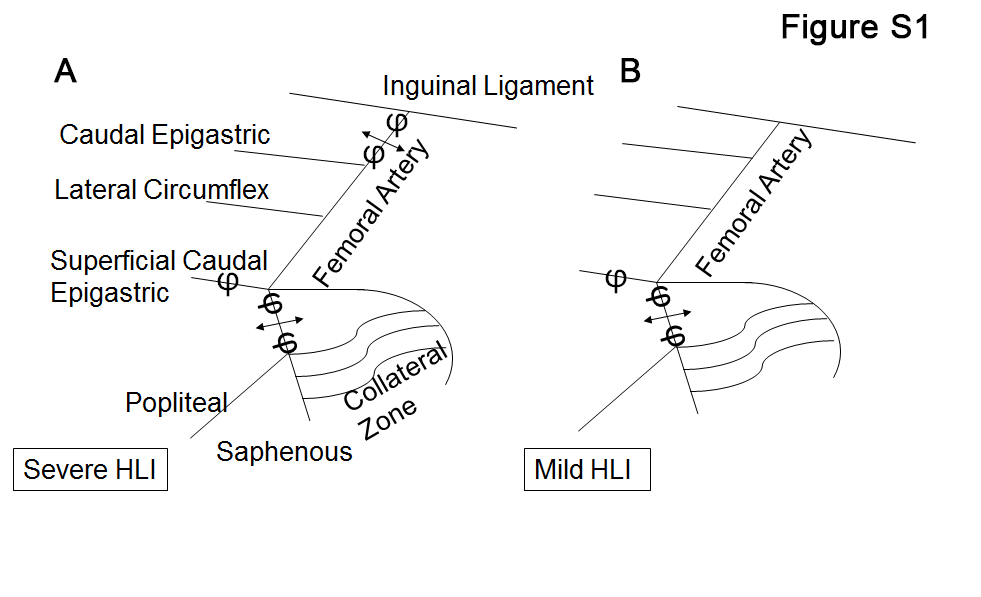

Supplement: Figure S1 — Schematic of surgical procedures used in the study. φ denotes ligation sites and ↔ denotes cutting sites. A) Severe HLI model involves ligation of the femoral artery distal to the inguinal ligament and proximal to the bifurcation of the popliteal artery. The artery was transected between the ligation points; in addition the superficial epigastric artery was ligated. B) Mild HLI model consisting of ligation and transection between the lateral caudal femoral artery and proximal to the bifurcation of the popliteal artery, plus ligation of the superficial epigastric. (TIF) [file pone.0112239.s001.tif]

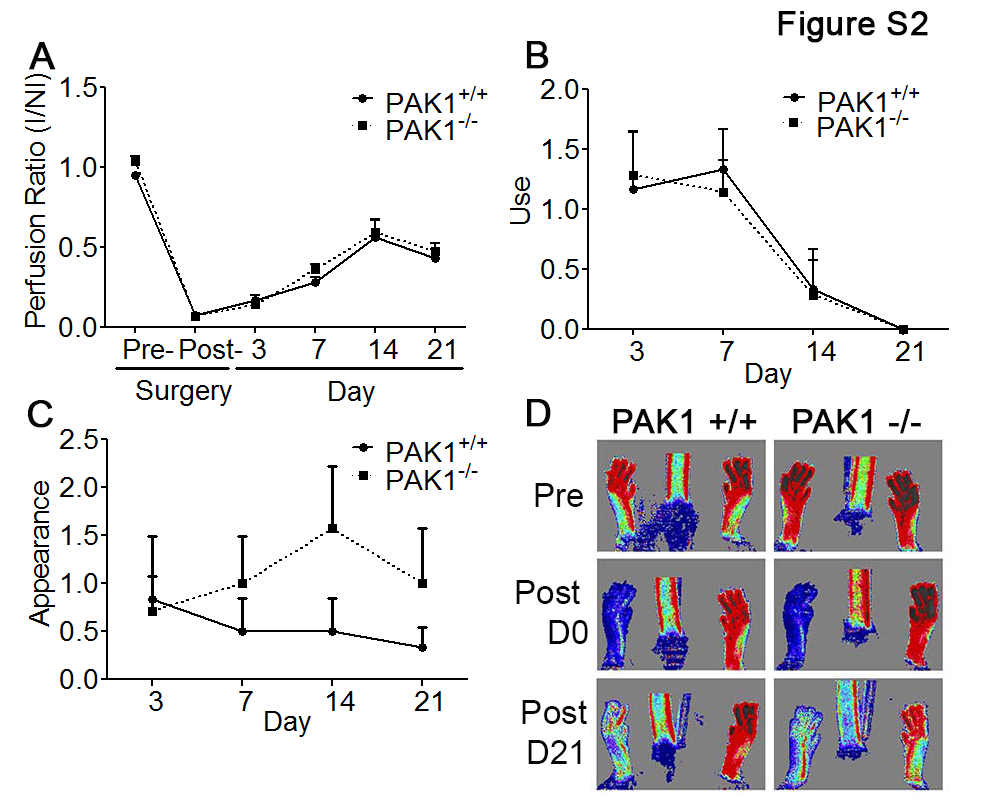

Supplement: Figure S2 — PAK1−/− neovascularization is unimpaired following mild HLI. A and D) I/NI perfusion ratios are almost identical after mild HLI in PAK1+/+ and PAK1−/− mice. B–C) Use and appearance scores that are not statistically different are in agreement with the perfusion ratio observation and confirm the lack of difference in ischemic limb function between PAK1−/− and PAK1+/+ mice (n = 7 for PAK1+/+ and n = 10 for PAK1−/−). (TIF) [file pone.0112239.s002.tif]
